# Retraction Note: *miR-874* inhibits gastric cancer cell proliferation by targeting *SPAG9*

**DOI:** 10.1186/s12885-025-15199-1

**Published:** 2025-10-27

**Authors:** Qin Hui Sun, Zong Xiu Yin, Zhi Li, Shu Bo Tian, Hong Chang Wang, Fang Xu Zhang, Le Ping Li, Chun Ning Zheng, Shuai Kong

**Affiliations:** 1 Department of Gastrointestinal Surgery, Shandong Provincial Hospital, Cheeloo College of Medicine, Shandong University, Jinan, 250021 China; 2 Department of Respiration Medicine, Jinan Central Hospital Affiliated to Shandong University, Jinan, 250013 China; 3 Department of Operating Room, Jinan Central Hospital Affiliated to Shandong University, Jinan, 250013 China; 4 Department of Gastrointestinal Surgery, Shandong Provincial Hospital Affiliated to Shandong First Medical University, Jingwu Road No.324, Jinan, 250021 China; 5 School of Clinical Medicine, Weifang Medical University, Weifang, 261042 China

**Retraction Note: BMC Cancer 20, 522 (2020)**

**https://doi.org/10.1186/s12885-020–06994-z**

The Editor has retracted this article after concerns over image integrity, specifically:

- Fig. 2c (NC-BGC-823), (miR-874 inhibitor –MGC-803), (miR-874 inhibitor – MKN-74) and Fig. 5c (SPAG9) in this article appear to overlap with each other.
- Fig. 2c (miR-874 inhibitor – BGC-823) in this article appears to overlap with Fig. 5A (DMSO-PANC-1) in a previously-published article by different authors [1].
- Fig. 5c (SPAG9 + miR-874) in this article appears to overlap with to Fig. 5A (Emodin-PANC1) and (DMSO-BxPC-3) in [1].

The authors have not been able to provide the original raw images. Therefore, the Editor has lost confidence in the data and conclusions of this article.

All authors agree with this retraction.
